# Burnout in Embryologists Working in Assisted Reproduction: Data from a Cross-Sectional Worldwide Study

**DOI:** 10.5935/1518-0557.20260058

**Published:** 2026

**Authors:** Raquel Urteaga, Amelia Díaz

**Affiliations:** 1 Faculty of Psychology, University of Valencia, Valencia, Spain

**Keywords:** burnout, embryologists, assisted reproduc-tion, worldwide study, gender

## Abstract

**Objectives:**

Recent studies have highlighted the concerning prevalence of burnout among embryologists working in assisted reproduction (AR), with embryologists showing the highest levels among AR professionals. How-ever, data on burnout among these professionals across different world regions remain very limited. Therefore, this study aimed to assess burnout levels among embryologists across World Health Organization regions, with compari-sons among four regions.

**Methods:**

This was a cross-sectional study. Burnout was assessed using a worldwide online survey completed by 759 respondents from 74 countries. Participants com-pleted the 15-item Maslach Burnout Inventory-General Survey, which assesses emotional exhaustion, depersonal-ization, and lack of personal accomplishment.

**Results:**

More than 80% of embryologists showed moderate or high levels of emotional exhaustion and de-personalization, whereas lack of personal accomplishment was present in a lower, although still substantial, propor-tion (67%). The comparison of means among Europe, Lat-in America, North America, and the Western Pacific showed only one significant difference: embryologists working in Europe had higher lack of personal accomplishment than those working in Latin America. However, significant differ-ences were found in the distribution of low, moderate, and high levels for all three burnout dimensions across world regions. Gender should be considered when studying burn-out in embryologists, as 97.8% of embryologists with high levels in all three burnout dimensions were women.

**Conclusion:**

Burnout is a serious global problem among embryologists working in AR. Future studies should investigate workplace stressors and preventive strategies to prevent and mitigate burnout, and should consider gen-der as an important variable.

## INTRODUCTION

Reproductive medicine is a highly specialized healthcare field that requires continuous training because of rapid scientific advances, the constant support needed by infertile couples, and the seamless coordination required among multidisciplinary team members to achieve optimal outcomes. Until a few years ago, the occupational well-being of these professionals was not a major focus of scientific research (Priddle *et al*., 2022). However, in recent years, numerous studies have increasingly sought to measure and evaluate the emotional status of these professionals, with growing concern about both their own well-being and the potential consequences of emotional distress within teams for patient outcomes (Govardhan *et al*., 2012; Kasraie & Kennedy, 2024; Rausch & Braverman, 2020).

Since Freudenberger (1974) defined burnout as a psychological state characterized by fatigue and frustration resulting from unmet professional expectations, commonly observed among care service workers, several definitions have refined this concept. Undoubtedly, the most widely used definition remains that of Maslach (1976), who described burnout as a syndrome characterized by emotional exhaustion, depersonalization, and reduced personal accomplishment at work, occurring among individuals whose daily tasks involve serving others. Emotional exhaustion refers to the depletion of emotional resources and the feeling of having nothing left to offer others. Depersonalization is characterized by emotional hardening and by negative, cynical, cold, and distant attitudes. Finally, lack of personal accomplishment is defined by feelings of inadequacy, perceptions of low competence, reduced professional effectiveness, unmet personal expectations, negative self-evaluation, and a lack of personal achievement. These three dimensions, widely adopted in the scientific literature, have since been used to measure and categorize burnout across professional groups (Faúndez, 2017). In 2019, the WHO recognized burnout syndrome as an occupational phenomenon and included it in the International Classification of Diseases (ICD-11): “Burn-out is a syndrome conceptualized as resulting from chronic workplace stress that has not been successfully managed. It is characterized by three dimensions: feelings of energy depletion or exhaustion; increased mental distance from one’s job, or feelings of negativism or cynicism related to one’s job; and reduced professional efficacy. Burn-out refers specifically to phenomena in the occupational context and should not be applied to describe experiences in other areas of life” (WHO, 2019).

Applied to assisted reproduction, a previous study conducted in 2024 (Urteaga & Díaz, 2024), including a Spanish sample of 380 healthcare professionals working in AR, found that approximately half of the sample exhibited signs of burnout across the three dimensions. Notably, embryologists were the most statistically affected professional group, with emotional exhaustion reaching 72.1%, depersonalization 48.1%, and lack of personal accomplishment 48.1%. Embryologists play a critical role in the success of AR procedures, performing complex tasks that require high precision and substantial responsibility, ranging from manual laboratory procedures to quality control and patient interaction (López-Lería *et al*., 2014). However, fertility care has traditionally focused on the mental health of patients, while the well-being of these professionals has been neglected until recent years, despite their fundamental role at the very origin of life (Peaston *et al*., 2022).

A close review of the scientific literature specifically focused on embryologists in human AR over the past five years shows recurring evidence of the severity of burnout syndrome and its threat to their mental health. For instance, Mittal *et al*. (2025) found that 74.97% of 787 embryologists in the Asia-Pacific region considered their role stressful, and only 38.5% felt comfortable discussing stress openly. Murphy *et al*. (2022) surveyed embryologists in the United States and found that 71% reported burnout symptoms in the exhaustion dimension, 63% in cynicism, and 54% reported being unable to cope with their workload. In a later study, Murphy *et al*. (2023) surveyed 104 embryologists in the United Kingdom and found that 60% reported symptoms of burnout in the exhaustion dimension, 42% in cynicism, and 68% felt unable to cope with their workload. Over one-third of professionals working in French assisted reproductive technology (ART) centers experienced job strain, a condition associated with increased risks of stress, burnout, and health problems (Delaroche *et al*., 2025). Finally, a national survey of Brazilian embryologists (Carneiro *et al*., 2025) found that 76.7% showed emotional exhaustion, 52.9% depersonalization, and 46.7% lack of personal accomplishment. All embryologists with high burnout levels in that study were women, considered their salary inadequate, and reported complete work-private life interference.

The reviewed literature shows high levels of burnout among embryologists working in AR, involving the three dimensions of emotional exhaustion, depersonalization or cynicism, and reduced personal accomplishment. Therefore, the aim of this study was to determine whether these high levels of burnout are common among embryologists working in AR across WHO regional zones (WHO, 2026): Europe, the Americas, the Western Pacific, Africa, the Eastern Mediterranean, and South-East Asia.

## MATERIALS AND METHODS

This cross-sectional study was conducted between November 5 and December 13, 2025, using the Survio platform.

### Procedure

The survey was distributed with the support of members of national and international scientific societies worldwide. The aim was to disseminate the survey link to as many embryologists as possible globally. Participants were presented with an information page describing the study’s ethics committee approval, anonymity, voluntary participation, study aim, and the informed consent required to take part. Permission to conduct the research was obtained from the Ethical Committee for Scientific Research of the authors’ university (Code: 3468621), and the study was conducted in accordance with the Declaration of Helsinki of 1975, as revised in 2013.

### Participants

A total of 759 responses were received from 74 countries. After applying the inclusion criterion of being an embryologist working in AR, the final sample comprised 742 participants. Regarding gender, 598 (80.1%) were women, 146 (19.7%) were men, and 2 (0.3%) identified as another gender. Age ranged from 22 to 68 years (M=40.59; SD=9.78; median=39). The sample was obtained through responses to a survey specifically designed for this purpose. The survey design incorporated information gathered during the first phase of the study, which consisted of a qualitative study based on semi-structured interviews conducted with an international expert panel of embryologists from five world regions: Europe, North America, Latin America, Asia, and Africa (Urteaga & Díaz, 2026a).

### Measures

The English version of the Maslach Burnout Inventory-General Survey (MBI-GS; Schaufeli *et al*., 1996) was used to measure burnout. The inventory contains 15 items rated on a 0-6 Likert scale (0=never, 1=a few times a year or less, 2=once a month or less, 3=a few times a month, 4=once a week, 5=a few times a week, 6=every day). The items are distributed across three subscales: emotional exhaustion, depersonalization, and lack of personal accomplishment. Higher scores indicate higher burnout levels. Subscale scores can be calculated using two methods. The first method sums the item scores for each subscale. The second method categorizes respondents into low, moderate, and high levels using the following cut-off points (Salanova *et al*., 2000): emotional exhaustion: low, 0 to 6; moderate, 7 to 14; high, 15 to 30; depersonalization: low, 0 to 2; moderate, 3 to 9; high, 10 to 24; lack of personal accomplishment: low, 0 to 22; moderate, 23 to 30; high, 31 to 36. Reliability in this study, assessed using Cronbach’s alpha, was adequate (0.93 for emotional exhaustion, 0.86 for depersonalization, and 0.91 for lack of personal accomplishment).

Additionally, the survey included questions about demographic variables such as gender and age.

### Statistical Analysis

First, skewness and kurtosis were calculated for the three variables studied: emotional exhaustion, depersonalization, and lack of personal accomplishment. Values between -2 and +2 were considered compatible with a normal distribution in the sample (George & Mallery, 2010; Hair *et al*., 2022). Second, means, standard deviations, and percentages in the low, moderate, and high levels of the three variables were obtained for the whole sample. Third, comparisons between WHO health regions were performed using one-way ANOVA and the Bonferroni post hoc test. However, because of the low number of participants from Africa, the Eastern Mediterranean, and South-East Asia (8, 9, and 8 participants, respectively), these regions were removed from the comparison, leaving Europe, the Americas, and the Western Pacific. For the analysis, the Americas were further divided into North America (Canada and the United States of America) and Latin America (Central and South America). The distribution of low, moderate, and high levels of the three burnout dimensions across the four world regions was compared using the chi-square test. Finally, gender comparisons in the four world regions were performed using ANOVA for Europe, which had a large sample, and the Mann-Whitney U test for the other regional samples. All analyses were performed using SPSS version 28 (IBM Corp., 2021).

## RESULTS

The Results section first presents findings for the whole sample and then findings by WHO region, with the Americas divided into North America and Latin America.

### Burnout dimensions in the whole sample

Table 1 shows the mean, standard deviation, number, and percentage of participants at the three burnout levels for the whole sample. Skewness and kurtosis were between -2 and +2, with values close to zero in all cases, supporting the assumption of normal distribution for these variables (George & Mallery, 2010; Hair *et al*., 2022). High levels of emotional exhaustion were found in 327 embryologists (44.1%), high levels of depersonalization in 279 (37.6%), and high levels of lack of personal accomplishment in 240 (32.3%). Although the percentages of high levels of emotional exhaustion, depersonalization, and lack of personal accomplishment were remarkable, when moderate levels were also considered, more than 80% of the sample showed concerning levels of emotional exhaustion and depersonalization, while lack of personal accomplishment was present in 67.1% of the sample.

**Table 1:** Skewness, kurtosis, range, mean, standard deviation, and number and percentage of participants in the burnout categories (low, moderate, and high) (N=742)

	Skewness	Kurtosis	Range	M	SD	Low n (%)	Medium n (%)	High n (%)
**Emotional exhaustion**	0.22	-0.80	0-30	13.91	7.65	144 (19.4)	271 (36.5)	327 (44.1)
**Depersonalization**	0.58	-0.53	0-24	8.19	6.06	148 (19.9)	315 (42.5)	279 (37.6)
**Lack of Personal Accomplishment**	0.51	-0.24	0-36	10.07	6.97	244 (32.9)	258 (34.8)	240 (32.3)

### Comparisons of burnout dimensions between Europe, Latin America, North America, and the Western Pacific

Table 2 presents the means, standard deviations, numbers, and percentages of embryologists in the low, moderate, and high burnout categories for Europe, Latin America, North America, and the Western Pacific. A one-way ANOVA was performed to compare the four regions across the three burnout dimensions. There was a significant difference in lack of personal accomplishment among the four regions analyzed, F(3,713)=3.87, *p*=0.009, eta^2^=0.16. Emotional exhaustion and depersonalization showed clear trends but did not reach statistical significance (F(3,713)=2.37, *p*=0.069, eta^2^=0.10; and F(3,713)=2.35, *p*=0.072, eta^2^=0.10, respectively). The assumption of homogeneity of variances was also met for all three dimensions (Levene F(3,713)=0.87, *p*=0.456 for emotional exhaustion; Levene F(3,713)=1.15, *p*=0.330 for depersonalization; and Levene F(3,713)=0.78, *p*=0.504 for lack of personal accomplishment).

**Table 2:** Means, standard deviations, and low, moderate, and high levels of emotional exhaustion, depersonalization, and lack of personal accomplishment in the four world regions (N=717)

		Emotional Exhaustion	Depersonalization	Lack of Personal Accomplishment
**Europe (n=499)**	M (SD)	13.77 (7.73)	8.55 (6.08)	10.51 (6.88)
	Low n (%)	99 (19.8)	88 (17.6)	150 (30.5)
	Moderate n (%)	188 (37.7)	216 (43.3)	172 (34.5)
	High n (%)	212 (42.5)	195 (39.1)	177 (35.5)
**Latin America (n=94)**	M (SD)	12.83 (8.02)	6.88 (6.34)	7.97 (7.33)
	Low n (%)	24 (25.5)	30 (31.9)	43 (45.7)
	Moderate n (%)	36 (38.3)	31 (33.0)	37 (39.4)
	High n (%)	34 (36.2)	33 (35.1)	14 (14.9)
**North America (n=54)**	M (SD)	14.41 (7.22)	8.46 (6.39)	10.87 (7.16)
	Low n (%)	9 (16.7)	13 (24.1)	17 (31.5)
	Moderate n (%)	17 (31.5)	21 (38.9)	13 (24.1)
	High n (%)	28 (51.9)	20 (37.0)	24 (44.4)
**Western Pacific (n=70)**	M (SD)	15.93 (7.06)	7.54 (5.20)	9.69 (6.36)
	Low n (%)	9 (12.9)	12 (17.1)	24 (34.3)
	Moderate n (%)	19 (27.1)	35 (50.0)	26 (37.1)
	High n (%)	42 (60.0)	23 (32.9)	20 (28.6)

Differences among the four world regions were further analyzed using Bonferroni post hoc tests. The results showed a significant difference between European and Latin American embryologists in lack of personal accomplishment (mean difference=2.54, *p*=0.007), with embryologists from Europe showing higher lack of personal accomplishment than those from Latin America. Three additional trends did not reach statistical significance: (1) emotional exhaustion differed between embryologists in Latin America and the Western Pacific region (mean difference=-3.10, *p*=0.064), with embryologists working in the Western Pacific showing higher emotional exhaustion than those working in Latin America; (2) depersonalization differed between embryologists in Europe and Latin America (mean difference=1.67, *p*=0.087), with embryologists working in Europe showing higher depersonalization than those working in Latin America; and (3) lack of personal accomplishment differed between embryologists in the Western Pacific and North America (mean difference=-2.90, *p*=0.085), with embryologists working in the Western Pacific showing lower lack of personal accomplishment than those working in North America.

Although the comparison of means among the four regions produced only one significant difference, in lack of personal accomplishment, the analysis of the distribution of low, moderate, and high burnout levels showed significant results for all three dimensions. Chi-square tests of independence were performed to examine the association between the three burnout dimensions and world region. Significant associations were found for emotional exhaustion, χ^2^(6,717)=12.46, *p*=0.050; depersonalization, χ^2^(6,717)=12.86, *p*=0.045; and lack of personal accomplishment, χ^2^(6,717)=21.42, *p*=0.002.

For emotional exhaustion, most embryologists in the four regions reported high levels, except in Latin America, where the percentage was higher at the moderate level than at the high level. Embryologists working in the Western Pacific region reported the highest percentage of high emotional exhaustion (60.0%), whereas embryologists in Latin America showed the lowest percentage (36.2%), with North America (51.9%) and Europe (42.5%) occupying intermediate positions. In addition, when the moderate and high categories were combined, emotional exhaustion affected more than 80% of embryologists working in the Western Pacific, North America, and Europe (87.1%, 83.4%, and 80.2%, respectively), whereas the corresponding percentage in Latin America was 74.5%.

In the depersonalization dimension, the percentages of embryologists from the four regions reporting high levels were similar, ranging from 32.9% to 39.1%. When the high and moderate levels were combined, more than 80% of embryologists in the Western Pacific and Europe were affected (82.9% and 82.4%, respectively). In North America, the corresponding percentage was 75.9%, while the lowest percentage was observed among Latin American embryologists (68.1%).

Finally, in the lack of personal accomplishment dimension, the number of embryologists reporting high levels differed considerably among the four regions. The highest percentage was observed among embryologists from North America (44.4%), followed by Europe (35.5%) and the Western Pacific (28.6%), whereas the lowest percentage was found in Latin America (14.9%). Lack of personal accomplishment presents a distinct pattern, as it seems to affect most embryologists working in North America and Europe, moderately affects those working in the Western Pacific, and affects those working in Latin America to a lesser extent. When moderate and high levels of lack of personal accomplishment were combined, there was only a small difference (less than 5%) between Europe, North America, and the Western Pacific (70.0%, 68.5%, and 65.7%, respectively), whereas the percentage for Latin America was 54.3%, highlighting a clear difference in this dimension.

Finally, gender among embryologists is a variable that has not received the attention it deserves. In our sample, more than 80% of embryologists were women, while men represented less than 20%. Gender analyses were performed in the four world regions, with no significant gender differences. Two trends were found in Europe and the Western Pacific, where women showed higher lack of personal accomplishment than men (Europe: men, mean=9.65; women, mean=10.72; t(496)=-1.43, *p*=0.076; Western Pacific: men, mean=3.00; women, mean=9.88; Mann-Whitney U=34.50, *p*=0.056). However, among the 46 participants from the four analyzed world regions who simultaneously reported high emotional exhaustion, depersonalization, and lack of personal accomplishment, 45 (97.8%) were women and 1 (2.2%) was a man.

## DISCUSSION

The aim of this study was to compare the levels of burnout dimensions found among embryologists in our sample with those reported in previous publications, both overall and by region: Europe, Latin America, North America, and the Western Pacific.

Previous studies have shown very high levels of emotional exhaustion among embryologists working in AR in different countries, including Spain, where the prevalence was 72.1% (Urteaga & Díaz, 2024), and Brazil, where it was 76% (Carneiro *et al*., 2025). Although the global percentage of embryologists reporting high emotional exhaustion in the present study was also high (44.1%), it was substantially lower than in those two studies. Emotional exhaustion was one of the most important dimensions across all four analyzed world regions. The Western Pacific reported the highest prevalence, affecting 60% of embryologists. In contrast, the percentage of embryologists experiencing high emotional exhaustion in Latin America was nearly half that figure, with a larger percentage of embryologists at a moderate level (38.3%) than at a high level (36.2%). Additionally, although the comparison of means revealed no significant differences among the four world regions, the comparison of the distribution of low, moderate, and high levels yielded significant results. This outlines a clear trend in which most professionals fall into the moderate and high categories. These findings confirm that emotional exhaustion is a defining characteristic of the workplace environment for embryologists across all four regions, particularly in Europe, North America, and the Western Pacific, and is less pronounced in Latin America. This regional variation may be related to workload differences. For instance, Kushnir *et al*. (2017) noted, in a review spanning 2004 to 2013, that Latin America had the lowest increase in demand for assisted reproduction during that period (16%), whereas regions such as Japan and Australia/New Zealand showed the highest increases in demand over the same period (82.6% and 76.3%, respectively). This relatively slow increase in demand in Latin America compared with the dramatic increase in the Western Pacific during the same period could explain the different exhaustion percentages between these regions.

Regarding North America, Murphy *et al*. (2022) found that 71% of U.S. embryologists reported high emotional exhaustion; in comparison, the present study, which also includes data from Canada, found a percentage of 51.9%, which was lower than expected. Meanwhile, the proportion of European embryologists with high emotional exhaustion (42.5%) was higher than the 35.4% reported in Spain by Urteaga & Díaz (2026b), but lower than the 60% observed among UK embryologists (Murphy *et al*., 2023). It should be noted that Urteaga & Díaz (2026b) separated burnout levels into high, moderate, and low categories, and the data reported correspond to the percentage of embryologists with high emotional exhaustion. However, the findings by Murphy *et al*. (2023) reported emotional exhaustion symptoms without reference to severity levels; therefore, that result may include moderate emotional exhaustion. When a similar approach was used in the present study, the percentages increased to 74.5% across all regions, confirming the high prevalence of emotional exhaustion among embryologists in the global regions studied.

The depersonalization dimension, also referred to as cynicism, showed the highest level of similarity across the four world regions compared. All regions fell within a high-level prevalence range of 33% to 39%. Although the comparison of means revealed no statistically significant differences between regions, comparing percentages across the three depersonalization levels revealed significant variation. Latin America had a similar distribution of embryologists across the three depersonalization levels, whereas the other regions-Europe, North America, and the Western Pacific-showed peak percentages at the moderate level, closely followed by the high level. Again, the percentages obtained in this study were lower than those reported in previous studies among embryologists, such as Spain with 42% to 48% (Urteaga & Díaz, 2024; Urteaga & Díaz, 2026b), the United Kingdom with 42% (Murphy *et al.,* 2023), the United States with 63% (Murphy *et al*., 2022), and Brazil with 52% (Carneiro *et al*., 2025). However, when the moderate level was included, the increase in percentages was substantial, reaching 82% in Europe and the Western Pacific, approximately 76% in North America, and 68% in Latin America, percentages higher than those found in the cited studies.

The dimension of reduced personal accomplishment showed high variability among the world regions studied. It was the only dimension with significant differences among regions, with embryologists working in Europe showing higher lack of personal accomplishment than those working in Latin America. Significant differences were also observed when the distribution of percentages across the three levels of lack of personal accomplishment was considered. North America showed the highest percentage of embryologists with high levels (44.4%), whereas Latin America showed the lowest percentage (14.9%); Europe and the Western Pacific were at intermediate levels, with 35.5% and 28.6%, respectively. These percentages were lower than those reported in previous studies (Carneiro *et al*., 2025; Murphy *et al*., 2022; 2023; Urteaga & Díaz, 2024; 2026b), but only when the high level alone was considered. When both high and moderate levels were combined, the resulting percentages were comparable to or even higher than those reported in previous national studies, ranging from 65% to 70% for Europe, North America, and the Western Pacific, while Latin American embryologists reported a non-negligible 54%.

Considering the three dimensions together, Latin America was the world region in which the percentage of embryologists presenting signs of burnout was lowest in our study. This may be related to the lower demand for AR in Latin America compared with other world regions (Kushnir *et al*., 2017). In a more recent publication by Peipert *et al*. (2023), a comparison of the 18 countries, including the United States, with the highest use of in vitro fertilization (IVF), the most common assisted reproduction technique, included no Latin American country, suggesting a lower level of assisted reproduction use in this region compared with other countries or world regions. One factor that could explain the lower use of IVF may be that the average maternal age at childbirth is lower than in other world regions, such as North America, Europe, and the Western Pacific, suggesting fewer difficulties when pregnancy is planned. Additionally, Baker *et al*. (2025), using data on the number of cycles conducted in 2017-2018 in different regions of the world, showed that the number of initiated cycles was ≥562,222 in Europe, 92,033 in North America, 43,138 in Australia and New Zealand, ≥870,587 in Asia, and 38,172 in Latin America. Considering that Australia, New Zealand, and parts of Asia are included in the Western Pacific region by the WHO, Latin America was the region where, at that time, the number of initiated cycles was lowest; this could explain the slightly lower percentages in the burnout dimensions found in this region. However, although the Baker *et al*. (2025) study is recent, it presents data from eight years ago, and there has been a steady increase in the use of AR in this region in later years, with 106,918 initiated cycles in 2019 and 127,351 in 2021 (Zegers-Hochschild *et al*., 2022; 2026). Consequently, cultural, organizational, and social factors may provide a better explanation for our results. García-Arroyo & Osca-Segovia (2018), in a meta-analysis on burnout in nine Latin American countries, proposed that the best explanation for differences between countries, with Brazil showing the highest burnout levels, could be found using cultural relativism theory and by highlighting cultural particularities.

In summary, the percentages of embryologists showing signs of burnout were high, and very high in the regions analyzed. Even the lowest percentages were above 50%, meaning that, even in the best-case scenario, one in two embryologists would exhibit signs of burnout. Therefore, it should be emphasized that burnout among embryologists is not country-specific but common across large areas of the world. Addressing this problem requires an in-depth analysis of the stressors contributing to burnout and the implementation of organizational and individual interventions to prevent and mitigate burnout.

One element seldom considered in most studies on burnout among embryologists is gender, which is usually treated only as a sample descriptor, perhaps because comparisons of means between men and women in the burnout dimensions have not shown significant differences. However, even considering that women are overrepresented in studies of embryologists working in AR (Murphy *et al*., 2022; Urteaga & Díaz, 2026b), this overrepresentation is even greater when focusing on the percentage of women who simultaneously show high levels in all three burnout dimensions: emotional exhaustion, depersonalization, and lack of personal accomplishment. This was the case in our sample, in which 97.8% of embryologists with high levels in all three dimensions were women, and it was also the case in the sample used by Carneiro *et al*. (2025), where all embryologists at these high levels were women. Overload, prolonged working hours, work-life imbalance, and many other stressors common in embryologists’ work (Astro *et al*., 2025; Basar & Duzcu, 2025) may affect women more than men. It should be considered that in many countries around the world, women carry a greater burden than men, mainly related to family care responsibilities (Molina, 2015; Mussida & Patimo, 2021).

This study has several limitations. First, the sample should be larger. Although country representation was broad, with 74 countries included, some countries had very few participants, which limited the analysis of other world regions. Second, because this was a cross-sectional study, causal relationships cannot be inferred. Third, the use of self-report measures is associated with biases such as acquiescence and social desirability. Future research should include longitudinal designs when the aim is to identify causal relationships. The role of gender may affect results and should be included in future analyses.

## CONCLUSION

This study assessed burnout among embryologists working in AR worldwide, including comparisons among Europe, Latin America, North America, and the Western Pacific. The results show that burnout is a common issue among these professionals, with few differences between regions and high percentages of embryologists at moderate and high levels in the three burnout dimensions: emotional exhaustion, depersonalization, and lack of personal accomplishment. Latin America appeared to have slightly lower levels than the other regions, although signs of burnout were still present. Finally, considering that women are overrepresented in the profession, gender should be considered in future research on this subject.

## References

[r1] Astro G, Gatti M, Costa M, Cosmelli A, Alteri A, Anastasi A, Cimadomo D, Santis L, Klinger FG, Licata E, Fernandez LS, Tomasi G, Vegni E, Sáiz IC, Prados N, Pisaturo V. (2025). Perceived workplace stressors and professional experiences of
clinical embryologists working in Italy and Spain: a pilot qualitative
study. Front Public Health.

[r2] Baker VL, Dyer S, Chambers GM, Keller E, Banker M, de Mouzon J, Elgindy E, Bai FM, Ishihara O, Jwa SC, Kupka MS, Zegers-Hochschild F, Adamson GD. (2025). International Committee for Monitoring Assisted Reproductive
Technologies (ICMART): world report for cycles conducted in
2017-2018. Hum Reprod.

[r3] Basar M, Duzcu T. (2025). The burnout: a silent saboteur in in vitro fertilization
laboratories. J Assist Reprod Genet.

[r4] Carneiro MM, Prates N, Guimaraes Io Tuco E, Santos C, Sampaio M, Moreira Alvarenga D, Goulart XAC, de Lima Bossi R (2025). Under pressure: Burnout among Brazilian embryologists. Results
from a nationwide survey. Fertil Steril.

[r5] Delaroche L, Bazin F, Sermondade N. (2025). Over one-third of professionals in French ART centers experience
job strain. Sci Rep.

[r6] Faúndez VO. (2017). Laudatio: Dra. Christina Maslach, Comprendiendo el
burnout. Cienc Trab.

[r7] Freudenberger HJ. (1974). Staff burn-out. J Soc Issues.

[r8] García-Arroyo J, Osca Segovia A. (2018). Effect sizes and cut-off points: a meta-analytical review of
burnout in latin American countries. Psychol Health Med.

[r9] George D, Mallery P. (2010). SPSS for Windows Step by Step: A Simple Guide and Reference 17.0
Update.

[r10] Govardhan LM, Pinelli V, Schnatz PF. (2012). Burnout, depression and job satisfaction in obstetrics and
gynecology residents. Conn Med.

[r11] Hair JF, Hult GTM, Ringle CM, Sarstedt MA. (2022). A Primer on Partial Least Squares Structural Equation Modeling
(PLS-SEM).

[r12] IBM Corp (2021). IBM SPSS Statistics for Windows (Version 28.0.0.0) [Computer
software].

[r13] Kasraie J, Kennedy H. (2024). Best practice for embryology staffing in HFEA licensed assisted
conception centres-guidance from Association of Reproductive & Clinical
Scientists. Hum Fertil (Camb).

[r14] Kushnir VA, Barad DH, Albertini DF, Darmon SK, Gleicher N. (2017). Systematic review of worldwide trends in assisted reproductive
technology 2004-2013. Reprod Biol Endocrinol.

[r15] López-Lería B, Jimena P, Clavero A, Gonzalvo MC, Carrillo S, Serrano M, López-Regalado ML, Olvera C, Martínez L, Castilla JA. (2014). Embryologists’ health: a nationwide online
questionnaire. J Assist Reprod Genet.

[r16] Maslach C. (1976). Burnout. Hum Behav.

[r17] Mittal G, Mantravadi KC, Malhotra K, Liow S, Ezoe K, Choy KW, Chang TA, Chung MK, Rose R, Chi L. (2025). The pipette and the pressure: Assessing stress levels in
embryologists special interest group-embryology, ASPIRE. Fertil Reprod.

[r18] Molina JA. (2015). Caring within the family: Reconciling work and family
life. J Fam Econ Issues.

[r19] Murphy A, Baltimore H, Lapczynski MS, Proctor G, Meyer EC, Glynn T, Domar AD, Collins MG. (2022). Embryologist burnout: Physical and psychological symptoms and
occupational challenges currently reported by U.S.
embryologists. Fertil Steril.

[r20] Murphy A, Lapczynski M, Proctor G, Meyer EC, Glynn T, Domar A, Gameiro S, Palmer G, Collins MG. (2023). The occupational challenges reported by UK embryologists: Stress,
fatigue, and burnout. Hum Reprod.

[r21] Mussida C, Patimo R. (2021). Women’s Family Care Responsibilities, Employment and Health: A
Tale of Two Countries. J Fam Econ Issues.

[r22] Peaston G, Subramanian V, Brunckhorst O, Sarris I, Ahmed K. (2022). The impact of emotional health on assisted reproductive
technology outcomes: a systematic review and meta-analysis. Hum Fertil (Camb).

[r23] Peipert BJ, Adashi EY, Penzias A, Jain T. (2023). Global in vitro fertilization utilization: How does the United
States compare?. F S Rep.

[r24] Priddle H, Pickup S, Hayes C, Association of Reproductive and Clinical Scientists (ARCS) (2022). Occupational health issues experienced by UK embryologists:
informing improvements in clinical reproductive science
practice. Hum Fertil (Camb).

[r25] Schaufeli WB, Leiter MP, Maslach C, Jackson SE., Maslach C, Jackson SE, Leiter MP (1996). The Maslach Burnout Inventory-Test Manual.

[r26] Rausch DT, Braverman AM. (2020). Burnout rates among reproductive endocrinology nurses: The role
of personality and infertility attitudes. Fertil Steril.

[r27] Salanova M, Schaufeli WB, Llorens S, Peiró JM, Grau R. (2000). Desde el “burnout” al “engagement”: ¿una nueva
perpectiva?. Rev Psicol Trab Org.

[r28] Urteaga R, Díaz A. (2024). Burnout in Assisted Reproduction Professionals: The Influence of
Stressors in the Workplace. Healthcare (Basel).

[r29] Urteaga R, Díaz A. (2026a). Burnout in embryologists from the perspective of an international
expert panel: A qualitative study. Behav Sci.

[r30] Urteaga R, Díaz A. (2026b). The Effect of Workplace Pressure and Experience on Burnout in
Embryologists Working in Assisted Reproduction in Spain. Int J Environ Res Public Health.

[r31] Zegers-Hochschild F, Crosby JA, Musri C, Souza MDCB, Martinez AG, Silva AA, Mojarra JM, Masoli D, Posada N, Reproduction LANOA. (2022). Assisted reproductive technologies in Latin America: the Latin
American Registry, 2019. JBRA Assist Reprod.

[r32] Zegers-Hochschild F, Crosby JA, Musri C, Petermann-Rocha F, Martinez G, Nakagawa H, Morente C, Palma-Govea A, Roque A, Latin American Network of Assisted Reproduction (2026). Assisted reproductive technology in Latin America: The Latin
American Registry, 2022. JBRA Assist Reprod.

[r33] WHO - World Health Organization (2019). World Health Assembly Update, 25 May 2019: International statistical
classification of diseases and related health problems (ICD-11).

[r34] WHO - World Health Organization (2026). Regional offices.

